# Association between energy intake patterns and outcome in US heart failure patients

**DOI:** 10.3389/fcvm.2022.1019797

**Published:** 2022-11-09

**Authors:** Zhang Fang, Zhe Wang, Xiaodi Cao, Ze-Mu Wang, Chuanchuan Yu, Weizhu Ju, Dianfu Li

**Affiliations:** ^1^Department of Cardiology, The First Affiliated Hospital of Nanjing Medical University, Nanjing, China; ^2^Department of Medical Statistics, School of Public Health, Sun Yat-sen University, Guangzhou, China

**Keywords:** heart failure, nutrient, dietary patterns, all-cause mortality, National Health and Nutrition Examination Survey

## Abstract

**Background:**

The association between dietary energy patterns, calories, and the outcomes of heart failure (HF) is still unclear.

**Objectives:**

To evaluate the proper energy intake patterns and daily calorie intake in patients with heart failure among US adults.

**Methods:**

The data were derived from the 2001–2014 National Health and Nutrition Examination Survey (NHANES). A calorie intake pattern variable was created using latent class analysis (LCA) based on the calorie ratio of three major nutrients. Cox proportional hazard regression models were used to evaluate the hazard ratios (HR) and 95% confidence intervals (CI) of the association between calorie intake and energy patterns. The primary endpoint was all-cause mortality.

**Results:**

Among 991 participants (mean age 67.3 ± 12.9 years; 55.7% men) who suffered from heart failure; the median calorie intake was 1,617 kcal/day [interquartile range (IQR): 1,222–2,154 kcal/day]. In the multivariable-adjusted model, moderate malnutrition was more frequent to death (HR: 2.15; 95% CI: 1.29–3.56). Low-carbohydrate pattern (LCP) and median-carbohydrate pattern (MCP) had lower risks of death compared to high-carbohydrate pattern (HCP) (LCP: HR: 0.76; 95% CI: 0.59–0.97; MCP: HR: 0.77; 95% CI: 0.60–0.98). No association between different amounts of calorie intake and all-cause mortality was found. There was an adjusted significant interaction between calorie intake and energy intake patterns (*p* = 0.019). There was a linear relationship between energy intake through HCP and all-cause mortality (*p* for non-linear = 0.557). A non-linear relationship between energy intake through MCP and all-cause mortality (*p* for non-linear = 0.008) was observed.

**Conclusion:**

Both LCP and MCP, compared to HCP, were associated with better outcomes in the HF population. The relationship between energy intake and all-cause death may be influenced by energy intake patterns in HF patients.

## Highlights

– Malnutrition is associated with poor outcomes in patients with heart failure (HF).
– No previous data exists about the dietary energy patterns and calorie intake in all-cause mortality of HF.
– Contrary to common belief, increasing the proportion of fat calories in the diet was associated with better outcomes in heart failure.
– This relationship between dietary energy patterns and the outcome was investigated further in the HF subgroup with different comorbidities.
– Daily calorie consumption affects heart failure outcomes based on dietary energy patterns of macro-nutrients.

## Introduction

Heart failure (HF) is currently a global public health problem, with high morbidity and mortality (1). In the United States, investigators estimated the prevalence of HF to be approximately 2.5% based on data reported in questionnaires, affecting nearly 6.5 million American adults and the prevalence of the disease continues rise (2). Despite promising advances in pharmacological treatment of HF (3), the outcome for HF patients remains unsatisfactory and its treatment is a long-term and expensive process. People with HF tend to have a poorer quality of life, and the disease itself can make patients frailer and more present with severe malnutrition, shortening survival times.

Nutrition is one of the modifiable factors of lifestyle and plays an important role in ensuring normal cardiac ejection fraction and maintaining favorable cardiac function (4). However, malnutrition in HF patients has been a common phenomenon partially because fluid and sodium restriction, which is an essential part of HF treatment, often leads to artificial reductions in active feeding and thus causes malnutrition, which is detrimental to patients with HF (5). There is currently limited evidence on the associated effects of nutritional interventions in patients with HF as evidence-based nutritional recommendations are lacking in major HF guidelines (3). In recent years, diet-related topics including calorie restriction (CR), dietary patterns, protein or amino acids supplementation and dietary fat intake have attracted extensive attention, such as the preventing and improving HF patient outcomes (6) and extending life expectancy (7) by calorie restriction. Mediterranean Diet (MedDiet) and the Dietary Approaches to Prevention of Hypertension (DASH) are the most widely studied dietary patterns in HF patients (8), but their focus is only on the intake of certain foods and nutrients (9), while lack of comprehensive consideration of calorie and associated energy intake patterns. Previous studies have shown that insufficient calorie intake was associated with poorer quality of life and greater burden of readmission in patients with HF (10) and adequate nutritional intake can delay the progression of HF (11). Therefore, nutritional assessment and related dietary interventions for HF patients are very necessary.

Considering that generally accepted nutritional strategies to improve quality of life and outcome in HF patients remain unmet, we designed this study to investigate the relationship between daily energy intake, different ratios of nutrient consumption, and all-cause mortality in HF patients, and explored the optimal calorie patterns for them.

## Materials and methods

### Study design

The data we examined were from the National Health and Nutrition Examination Survey (NHANES) 2001–2014 which is ongoing surveys of health status performed in 2-year cycles by the National Center for Health Statistics, Centers for Disease Control and Prevention. Its data were designed to determine the risk factors of diseases and to provide critical information on the health and nutritional status of the US population. The detailed survey operations manuals, consent documents, and brochures of NHANES can be viewed on the NHANES website. All data of this study are also publicly available at http://www.cdc.gov/nchs/nhanes.htm.

### Population

**FIGURE 1:**
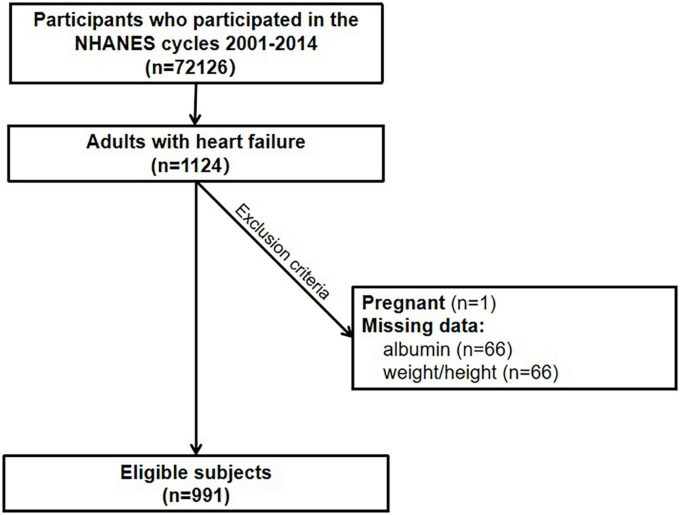
Flow diagram.

Our analyses were limited to NHANES 2001–2014 participants considering the consistency of variables. The flowchart of patient inclusion is shown in Figure 1. Participants aged 18 years and older who self-reported congestive HF and participated in a 24-h dietary recall assessment were included (*n* = 1,124). Further exclusions were made for those who had missing data on height and weight (*n* = 66), had missing serum albumin data needed to define nutritional risk index (NRI) (*n* = 66), and were pregnant (*n* = 1). After exclusions, 991 were used for this analysis. Data collection was reviewed and approved by the National Center for Health Statistics Research Ethics Review Board and signed informed consent forms were obtained from participants enrolled in the study.

### Dietary assessment

Daily dietary assessment was an NHANES-derived variable from Total Nutrient Intakes File, whose data were obtained through a 24-h Dietary Recall interview. The respondents needed to report all foods and beverages consumed during the previous 24 h. In the interview. A set of 3-dimensional measuring guides were used to help the respondent estimate the portion size. Information collected from the interview will be coded and linked to a database of nutrient composition of foods. The energy intake ratio of the three nutrients (fat, protein, and carbohydrate) was calculated by their intake. Diets were assessed in terms of total energy intake and calorie patterns. An overall calorie pattern variable was created using latent class analysis (LCA) based on proportion of energy of three major nutrients in total energy intake (each factor had three levels: lowest tertile, middle tertile, and highest tertile).

### Covariates

Demographic data were obtained through relevant questionnaires including gender, age, race, education, marital status, income, occupation, and type of health insurance. Participants were divided into three groups based on their poverty-to-income ratios: low (≤ 1), midrange (1–4), and high (≥ 4) (12). Less than a high school diploma, a high school graduate or its equivalent, and a college degree or more were the three categories for education. According to the commonly used socioeconomic index in the US, occupations were grouped into upper-skilled jobs (socioeconomic index 50), lower-skilled jobs (socioeconomic index 50, including retirees16 and students), and unemployed jobs (13). Health insurance was separated into three categories: private health insurance, public health insurance only, and no health insurance (14). We divided smoking status into former, current, and never. We also considered the exercise factor, but since the variables measuring exercise also varied in a different cycle, we calculated the metabolic equivalent at each cycle and divided the population into three groups based on the metabolic equivalent (15). The body mass index (BMI) is obtained through Body Measures File. We used the NRI to assess nutritional status. The NRI was calculated as NRI = (1.519 × serum albumin, g/dL) + [41.7 × weight (kg)/ideal body weight (IBW; kg)] (16). The IBW was calculated with the Lorentz equations (For men: H=100–[(H–150)/4], For women: H=100–[(H–150)/2.5]. NRI scores of > 100, 97.5–100, 83.5–97.5, and < 83.5 indicate no, mild, moderate, and severe risk of nutrition-related complications, respectively. We also included several comorbidities that may affect the outcome of HF, including hypertension, diabetes, stroke, myocardial infarction (MI), and chronic kidney disease (CKD). Diabetes was defined as fasting glucose of at least 7.0 mmol/L, non-fasting glucose of at least 11.1 mmol/L, glycated hemoglobin of at least 6.5%, use of glucose-lowering drugs, or self-reported diabetes. The history of the other above-mentioned diseases was obtained in the form of a self-report. For example, we defined CKD based on the answer to this question, “Have you ever been told by a doctor or other health professional that you had weak or failing kidneys? Do not include kidney stones, bladder infections, or incontinence?” To the question “Have you had shortness of breath either when hurrying on the level or walking up a slight hill?” Those who answered “yes” were defined as New York Classification of Cardiac Function (NYHA) III-IV of cardiac function. Meanwhile, the dietary inflammation index (DII) was calculated by dietary questionnaire (17).

### Ascertainment of mortality outcomes

The primary outcome was all-causes mortality, which was identified through linkage to the National Death Index (NDI) through December 31, 2015. The NDI is a centralized NCHS database of all deaths in the United States. Eligible participants were matched to this database to determine mortality status.

### Statistical analyses

Baseline characteristics for both groups were compared using the Wilcoxon rank sum test and Student’s *t*-test for continuous variables and the chi-square test for categorical variables. Multiple interpolations were used to deal with missing data on covariables. We used Cox proportional hazard regression models to estimate the hazard ratios (HR) and 95% confidence intervals (CI) associated with all-cause death and calorie intake pattern. We adjusted for sex, age, race, education, income, occupation, type of health insurance, marital status, smoking status, exercise, sodium intake, BMI, NRI, DII, comorbidities (hypertension, diabetes, MI, stroke, CKD), and NYHA Classification. We additionally fitted the restricted cubic spline with four knots at the 5th, 35th, 65th, and 95th to examine a linear relation between calorie intake and all-cause mortality in different patterns to explore the relevance. We performed a subgroup analysis based on comorbidities and calorie intake patterns. For database management and statistical analysis, we used R software, Version 4.1.1, and considered both two-sided *P*-values and P-interaction values < 0.05 to be significant.

## Results

### Population characteristics

**FIGURE 2:**
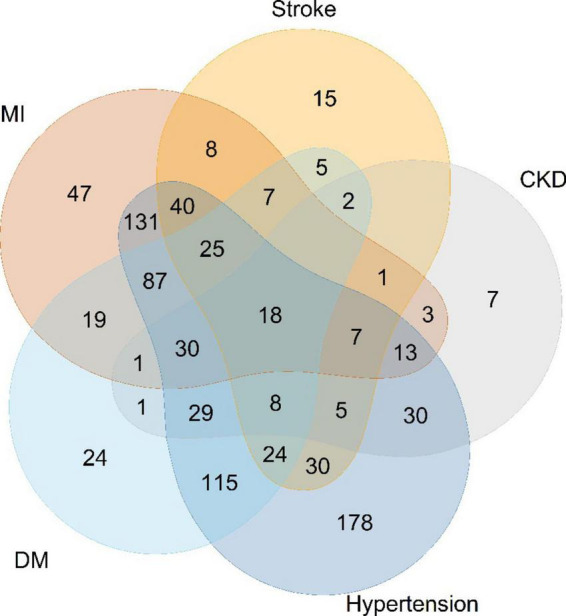
Numbers of heart-failure patients with different comorbidities. MI, myocardial infarction; CKD, chronic kidney diseases; DM, diabetes mellitus.

The baseline characteristics of the study are presented in Table 1. Among 991 participants from NHANES 2001–2014 (mean age 67.3 years, SE ± 12.9; 55.7% men), 590 (59.5%) were Non-Hispanic White. The median calorie intake was 1,617 kcal/day (IQR: 1,222–2,154 kcal/day). The number of HF patients with the underlying disease is shown in Figure 2. 88 patients with HF had no comorbidities 0.427 deaths were recorded during a mean follow-up of 67.5 months. There was no difference in HF death rates between men and women (*p* = 0.264). Living HF patients had higher BMI (*p* < 0.001), lower risk of malnutrition (*p* < 0.001), and more energy intake (*p* = 0.019) than those with primary outcomes. In terms of diet, the survivors ate more carbohydrates (*p* = 0.042) and sodium (*p* = 0.014).

**TABLE 1:** Baseline characteristics of the patients.

Patient characteristics	All	Missing	No event	Event	*P*-value
	*N* = 991		*N* = 564	*N* = 427	
Age, years	67.3 ± 12.9	0/991	62.9 ± 12.8	73.1 ± 10.5	<0.001
**Gender, %**		**0/991**			**0.264**
Men	552 (55.7)		305 (54.1)	247 (57.8)	
Women	439 (44.3)		259 (45.9)	180 (42.2)	
**Race, %**		**0/991**			**<0.001**
Mexican American	91 (9.18)		55 (9.8)	36 (8.4)	
Other Hispanic	51 (5.15)		39 (6.9)	12 (2.8)	
Non-Hispanic White	590 (59.5)		297 (52.7)	293 (68.6)	
Non-Hispanic Black	222 (22.4)		153 (27.1)	69 (16.2)	
Other race—including multi-racial	37 (3.73)		20 (3.5)	17 (4.0)	
**Marital status, %**		**0/991**			**<0.001**
Married	477 (48.1)		294 (52.1)	183 (42.9)	
Widowed/divorced/separated	414 (41.8)		198 (35.1)	216 (50.6)	
Not married	100 (10.1)		72 (12.8)	28 (6.6)	
**Income, %**		**67/991**			**0.008**
≤1.0	229 (24.8)		143 (27.1)	86 (21.7)	
1.0–4.0	574 (62.1)		305 (57.9)	269 (67.8)	
≥4.0	121 (13.1)		79 (15.0)	42 (10.6)	
**Medical insurance, %**		**2/991**			**<0.001**
No insurance	76 (7.68)		60 (10.7)	16 (3.7)	
Public insurance	800 (80.9)		424 (75.4)	376 (88.1)	
Private insurance	113 (11.4)		78 (13.9)	35 (8.2)	
**Occupation, %**		**45/991**			**<0.001**
Unemployment	327 (34.6)		210 (38.9)	117 (28.8)	
Lower-skilled	598 (63.2)		312 (57.8)	286 (70.4)	
Upper-skilled	21 (2.22)		18 (3.3)	3 (0.7)	
**Education, %**		**1/991**			**0.022**
No high school graduate	394 (39.8)		211 (37.4)	183 (43.0)	
High school graduate	239 (24.1)		129 (22.9)	110 (25.8)	
College or above	357 (36.1)		224 (39.7)	133 (31.2)	
**Exercise, %**		**0/991**			**<0.001**
Inactive	557 (56.2)		264 (46.8)	293 (68.6)	
Median	258 (26.0)		173 (30.7)	85 (19.9)	
Active	176 (17.8)		127 (22.5)	49 (11.5)	
**Smoke, %**		**0/991**			**0.006**
Never	382 (38.5)		220 (39.0)	162 (37.9)	
Former	411 (41.5)		214 (37.9)	197 (46.1)	
Current	198 (20.0)		130 (23.0)	68 (15.9)	
BMI, kg/m^2^	31.3 ± 7.8	0/991	32.3 ± 8.3	29.9 ± 7.0	<0.001
Current height, cm	167.0 ± 10.3	0/991	167.0 ± 10.5	166.1 ± 10.0	0.206
Current weight, kg	87.1 ± 24.0	0/991	90.3 ± 25.2	82.9 ± 21.5	<0.001
**Myocardial infarction, %**		**5/991**			**0.228**
No	553 (56.1)		325 (57.8)	228 (53.8)	
Yes	433 (43.9)		237 (42.2)	196 (46.2)	
**Stroke, %**		**3/991**			**0.042**
No	793 (80.3)		465 (82.6)	328 (77.2)	
Yes	195 (19.7)		98 (17.4)	97 (22.8)	
**Hypertension, %**		**1/991**			**0.324**
No	221 (22.3)		119 (21.1)	102 (23.9)	
Yes	769 (77.7)		445 (78.9)	324 (76.1)	
**Diabetes mellitus, %**		**33/991**			**0.04**
No	581 (60.6)		347 (63.6)	234 (56.8)	
Yes	377 (39.4)		199 (36.4)	178 (43.2)	
**CKD, %**		**2/991**			**0.172**
No	834 (84.3)		483 (85.8)	351 (82.4)	
Yes	155 (15.7)		80 (14.2)	75 (17.6)	
Albumin, g/L	40.6 (3.48)	0/991	40.94 (3.33)	40.07 (3.61)	<0.001
**NYHA, %**		**38/991**			**0.058**
1–2	262 (27.5)		160 (30.0)	102 (24.3)	
3–4	691 (72.5)		373 (70.0)	318 (75.7)	
**Malnutrition, %**		**0/991**			**<0.001**
No	945 (95.4)		551 (97.7)	394 (92.3)	
Mild,	18 (1.82)		7 (1.2)	11 (2.6)	
Moderate	28 (2.83)		6 (1.1)	22 (5.2)	
Total energy intake, kcal/day	1617.0 [1222.0, 2154.0]	0/991	1665.00 [1253.0, 2270.5]	1572.00 [1208.0, 2024.5]	0.019
Protein intake, g	62.9 [45.9, 86.2]	0/991	64.9 [46.3, 88.4]	61.1 [44.9, 82.6]	0.069
Carbohydrate intake, g	203.3 [143.0, 264.3]	0/991	209.5 [144.7, 280.3]	194.9 [141.7, 250.9]	0.042
Fat intake, g	60.2 [40.2, 86.7]	0/991	62.1 [40.7, 90.0]	57.9 [39.7, 80.6]	0.056
Sodium, mg	2565.0 [1874.5, 3637.0]	0/991	2689.5 [1926.5, 3781.0]	2508.0 [1809.0, 3400.5]	0.014
Dietary inflammation index	0.2 [-0.7, 1.6]	0/991	0.4 [-0.9, 1.7]	0.0 [-0.5, 1.3]	0.438

Values are mean ± SD, median (interquartile range) or *n* (%). CKD, chronic kidney diseases; NYHA, New York Heart Association; DII, dietary inflammation index.

### Calorie intake pattern

**FIGURE 3:**
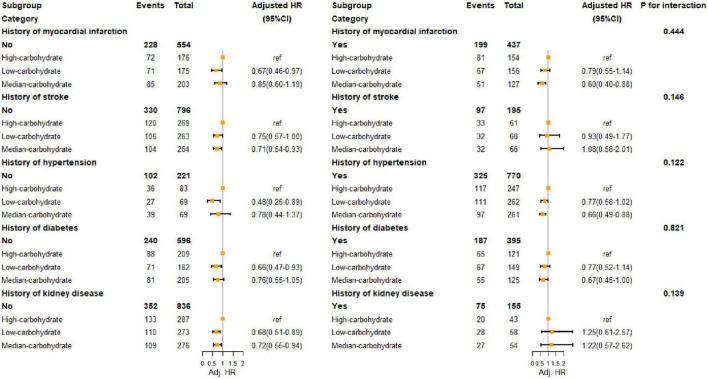
Hazard ratio of all-cause death of HF population with different comorbidities. MI, myocardial infarction; CKD, chronic kidney diseases.

We divided calorie intake patterns into three categories by LCA. The results of LCA were consistent with the proportion of carbohydrate energy classification by tertile. Patterns 1, 2, and 3 correspond to the lowest tertile (≤ 46.0%), medium tertile (46.0–54.4), and highest tertile (> 54.4%) groups of carbohydrate intake, respectively. The proportion of fat energy in pattern 1 exceeding 37.2 was 75.5%. In pattern 3, the majority (78.5%) of the population received less than 30.5% of their total energy from fat. Therefore, we defined patterns 1, 2, and 3 as, respectively, low-carbohydrate pattern (LCP), median-carbohydrate pattern (MCP), and high-carbohydrate pattern (HCP). LCP and MCP had lower risks of death compared to HCP (LCP: HR: 0.76; 95% CI: 0.59–0.97; MCP: HR: 0.77; 95% CI: 0.60–0.98) in Model 1. After we did a sensitivity analysis that excluded people with cancer, LCP was associated with a lower risk of death (HR: 0.72; 95% CI: 0.54–0.97). We examined interactions and performed subgroup analyses in subgroups with other diseases, and the results are shown in Figure 3 and Supplementary Table 1. Among people with a history of MI, diabetes, or hypertension, MCP showed better outcomes, the same as people without stroke. However, LCP was associated with a lower risk in people without comorbidities. There was no interaction between calorie intake patterns and diseases.

### Calorie intake

**FIGURE 4:**
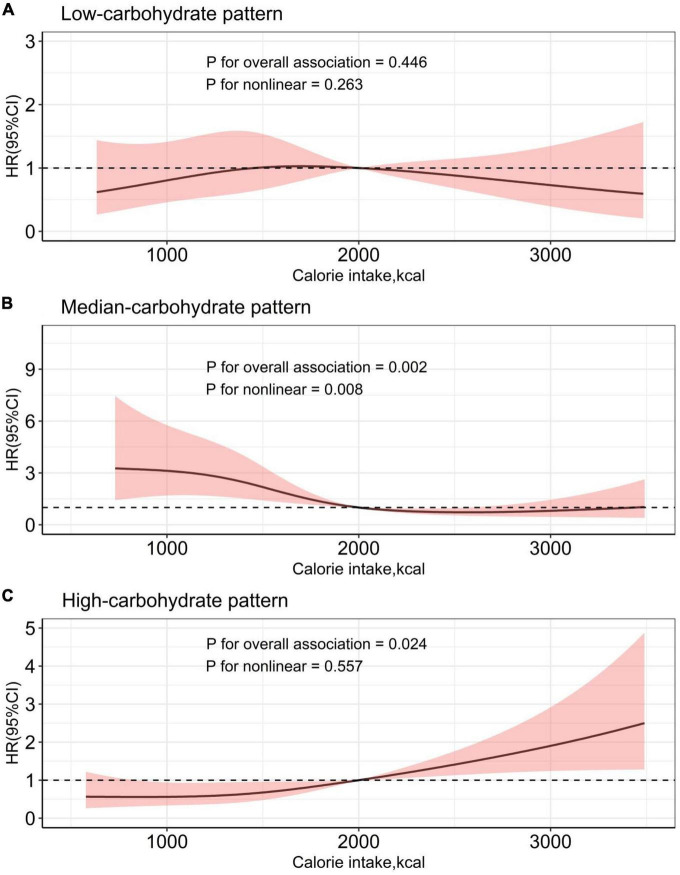
Calorie intake and risk for all-cause mortality of heart failure in different dietary patterns.

We found that poorer nutritional status, assessed by NRI, was associated with all-cause mortality in HF in Model 1 (Mild malnutrition: HR: 2.01; 95% CI: 1.03–3.90; Moderate malnutrition: HR: 2.15; 95% CI: 1.29–3.56) as seem in Table 2, After multivariable adjustment. We did not observe any association between different amounts of calorie intake and all-cause mortality. There was an adjusted significant interaction between calorie intake and energy intake patterns (*p* = 0.019). We performed a subgroup analysis based on calorie intake patterns shown in Table 3, a higher risk of death was shown in the third and fourth quartile compared with the second quartile (> 1222.0 to ≤1627.0 kcal/d) in HF patients with HCP. As shown in Figure 4, We found a linear relationship between energy intake through HCP and all-cause mortality (p for non-linear = 0.557), We also found a non-linear relationship between energy intake through MCP and all-cause mortality (*p* for non-linear = 0.008), with the lowest risk occurring at 2564.9 kcal. In MCP, the fourth quartiles (HR: 0.29; 95% CI: 0.14–0.61) were linked to lower risk of all-cause death. We observed no significant correlation between calorie intake and death in LCP (p for overall association = 0.446).

**TABLE 2:** Hazard ratio of outcomes by adjusted analysis.

	Model 1	
	HR (95%CI)	*P*-value
Age, years	1.07 (1.06–1.09)	<0.001
Women	0.54 (0.42–0.70)	<0.001
**Race**		
Mexican American	Ref	
Other Hispanic	0.72 (0.37–1.42)	0.347
Non-Hispanic White	1.01 (0.69–1.49)	0.957
Non-Hispanic Black	0.85 (0.55–1.30)	0.454
Other race—including multi-racial	1.22 (0.66–2.23)	0.529
**Marital status**		
Married	Ref	
Widowed/divorced/separated	1.37 (1.10–1.72)	0.006
Not married	1.91 (1.23–2.96)	0.004
**Income**		
≤1.0	Ref	
1.0–4.0	1.06 (0.82–1.37)	0.671
≥4.0	0.68 (0.45–1.04)	0.073
**Medical insurance**		
No insurance	Ref	
Public insurance	1.48 (0.87–2.5)	0.149
Private insurance	1.80 (0.97–3.35)	0.063
**Occupation**		
Unemployment	Ref	
Lower-skilled	0.80 (0.63–1.02)	0.075
Upper-skilled	0.84 (0.36–1.99)	0.698
**Education**		
No high school graduate	Ref	
High school graduate	1.43 (1.11–1.83)	0.005
College or above	1.07 (0.84–1.36)	0.602
**Smoke**		
Never	Ref	
Former	0.95 (0.75–1.20)	0.671
Current	1.00 (0.72–1.39)	0.987
**Exercise**		
Low	Ref	
Median	0.61 (0.48–0.79)	<0.001
High	0.58 (0.42–0.80)	0.001
Myocardial infarction	0.91 (0.75–1.12)	0.384
Diabetes mellitus	1.45 (1.17–1.81)	0.001
Stroke	1.08 (0.85–1.38)	0.528
Hypertension	0.86 (0.67–1.09)	0.202
Kidney failure	1.29 (0.98–1.68)	0.066
NYHAIII-IV	1.45 (1.15–1.85)	0.002
**BMI**		
<25	Ref	
25–30	0.72 (0.54–0.98)	0.033
30–40	0.85 (0.63–1.16)	0.305
≥40	0.89 (0.58–1.37)	0.591
**Malnutrition**		
No	Ref	
Mild	2.01 (1.03–3.90)	0.040
Moderate	2.15 (1.29–3.56)	0.003
DII	1.02 (0.96–1.09)	0.448
**Dietary pattern**		
High-carbohydrate pattern	Ref	
Low-carbohydrate pattern	0.76 (0.59–0.97)	0.028
Median-carbohydrate pattern	0.77 (0.60–0.98)	0.035
**Total energy intake**		
Q1	0.94 (0.72–1.24)	0.683
Q2	Ref	
Q3	1.07 (0.81–1.42)	0.640
Q4	0.98 (0.68–1.40)	0.910

Values are *n* or HR (95% CI). CI, confidence interval; HR, hazard ratio; other abbreviations as in Table 1.

**TABLE 3:** Hazard ratio of all-cause death based on dietary patterns.

Dietary pattern	Total energy intake	HR (95%CI)	*P*-value
Low-carbohydrate	Q1	1.44 (0.85–2.44)	0.172
	Q2	Ref	0.172
	Q3	0.65 (0.38–1.11)	0.111
	Q4	0.29 (0.14–0.61)	0.001
Median-carbohydrate	Q1	0.60 (0.35–1.02)	0.057
	Q2	Ref	
	Q3	0.77 (0.44–1.35)	0.363
	Q4	0.93 (0.48–1.78)	0.819
High-carbohydrate	Q1	1.37 (0.82–2.29)	0.224
	Q2	Ref	
	Q3	2.22 (1.32–3.74)	0.003
	Q4	2.14 (1.09–4.20)	0.026

Subgroup analysis of adjusting for sex, age, race, socioeconomic status, marital status, smoking status, exercise, body mass index, nutritional risk index, dietary inflammation index, comorbidities (hypertension, diabetes, myocardial infarction, stroke, chronic kidney diseases) and New York Classification of Cardiac Function.

## Discussion

Malnutrition was associated with higher all-cause mortality in HF patients. Our study found that different energy intake patterns were associated with the outcome of HF patients. LCP (≤ 46.0% of energy from carbohydrate) or MCP (46.0–54.4% of energy from carbohydrate) have a better outcome and HCP (> 54.4% of energy from carbohydrate) has a more unfavorable outcome among the patients with HF. We also found that the relationship between energy intake and all-cause death may be influenced by energy intake patterns in HF patients.

A recent analysis of calorie intake patterns and adverse outcomes in the general population showed a U-shaped curve, with the lowest risk of all-cause mortality in the population consuming calorie patterns that intake 50–55% of carbohydrates, while the low (< 40%) and high (> 70%) carbohydrate calorie patterns were all associated with an increased risk of death (18). However, several studies have shown that low-carbohydrate-diet scores not associated with increased risks of coronary heart disease or total mortality, which depended on the quality and food sources of macronutrients (19, 20). However, there is little evidence that similar result can be generalized to patients with HF, because such patients often have intestinal function changes (21) and metabolic impairment (22), which may disorganize the absorption and utilization of energy. In our study, LCP with high fat and protein proportion was more likely to offer a better outcome for HF patients when using HCP as a reference. These findings suggested that patients with HF might benefit from increased fat intake. Previous studies have also shown that low-fat diets didn’t reduce morbidity or mortality from cardiometabolic diseases and might not be used for the prevention of these diseases (23, 24). And chronic heart failure patients with low cholesterol were instead associated with increased mortality (25, 26). What’s worse, low-fat diets are often accompanied by a high intake of carbohydrates to make up for the loss of energy. While the pro-inflammatory effects of carbohydrates may lead to a systemic inflammatory response (27).

HF is precisely a metabolic disease and a systemic inflammatory response (28). This may be related to the propensity of the heart to consume substrates for energy under pathological conditions, influenced by the etiology of HF and other comorbidities (29). As more efficient substrates in cardiometabolic processes, ketone bodies are elevated in HF patients and serve as an alternative fuel for increased “productivity” (30–33). We advocate low-carb diets because low carbohydrate intake favors ketone body production and utilization to compensate for the reduction in cardiac glucose and fatty acid oxidation (34), rather than increased cardiomyocyte oxidation of fatty acids (35). Moreover, recent researches suggested that SGLT-2 inhibitors might help maintain higher ketone body levels in the body (31, 36–38), which might be related to its cardioprotective effects. In addition, ischemic heart disease and hypertension are major contributors to HF, in which ketone body utilization-related enzymes increased as well (33, 34, 39). Our study found that the MCP was indeed beneficial in patients with hypertension and coronary heart disease, while the advantages of the LCP seemed to be attenuated, which might be due to the adverse effects of high fat intake on comorbidities. Further studies are still needed to understand the underlying molecular mechanisms of these effects.

In addition, we explored the relationship between total energy intake and outcome in patients with HF based on different energy intake distributions. We found that this relationship seemed to be affected by the form of energy supplements, which required us to consider the allocation of energy sources when providing energy supplements to the population with heart failure. Calorie restriction (CR) is a widely studied dietary intervention in the field of HF treatment with many positive effects (40), including reduction of left ventricular hypertrophy (41), reduction of myocardial ischemic injury to improvement variable reserve (42), and improving cardiac function (6). Our research shed light on the exact energy intake pattern of nutrient proportion that might benefit from CR. Additionally, more calorie intake seemed to have better outcomes in MCP, compensating for the advantage of CR. And more notably, more than one-fifth of our study population suffered from the complications of malnutrition. Insufficient calorie intake may cause poorer quality of life and increased risks of readmission in this group of people. We provide potential evidence for the calorie intake of HF people who are malnourished or who need to consume more energy.

### Study limitations

Dietary alterations may exist after assessment. Different dietary treatments may be required for the diverse etiology of HF, which is not exactly clear in our study. Also, our failure to classify HF in line with ejection fraction may need to be remedied in future studies.

## Conclusion

In this study, low carbohydrate patterns and median carbohydrate patterns were associated with lower total mortality. We observed a U-shaped relationship between energy intake and mortality, under the median carbohydrate pattern. While in the high carbohydrate patterns, the more the intake of energy was, the worse the outcome was. These findings suggested that the associations of energy intake with mortality may depend on the energy intake patterns in HF patients.

## Perspectives

### Competency in medical knowledge

The outcome of HF is influenced by nutritional status and energy intake patterns, and low-carb proportion diets may assist improve the outcome of HF. Whether increase daily calorie intake or reduce it depends on different percentages of nutrients consumption. Nutritional assessment and dietary therapy have potential prognostic value in HF patients.

### Translational outlook

Possible approaches were provided for selecting suitable patients for dietary intervention, which have significant implications for ameliorating adverse outcomes of HF. Additional researches are needed to expose the pathophysiological mechanisms underlying the relationship between various energy patterns, calorie consumption, and outcome of HF.

## Data availability statement

The raw data supporting the conclusions of this article will be made available by the authors, without undue reservation.

## Ethics statement

The studies involving human participants were reviewed and approved by the National Center for Health Statistics, Centers for Disease Control and Prevention. The patients/participants provided their written informed consent to participate in this study. Written informed consent was obtained from the individual(s) for the publication of any potentially identifiable images or data included in this article.

## Author contributions

DL conceived and designed the study. WJ conceived the study. ZW and CY analyzed the data. ZF, XC, and Z-MW wrote the manuscript. All authors provided critical revisions of the manuscript and approved the final manuscript.

## Supplementary material

Supplementary Table 1 Hazard ratio of all-cause death of HF population with different comorbidities. MI, myocardial infarction; CKD, chronic kidney diseases.

## Abbreviations

BMI: body mass index
CR: calorie restriction
DII: dietary inflammation index
HCP: high-carbohydrate pattern
HF: heart failure
LCP: low-carbohydrate pattern
MCP: median-carbohydrate pattern
NRI: Nutritional Risk Index.
